# Novel Suicide by Division of a Chronically Infected, Externalised Axillofemoral Graft Presenting Challenges in Prehospital Assessment of Mental Capacity

**DOI:** 10.1155/2012/795648

**Published:** 2012-03-18

**Authors:** Lewis G. Stevens, Keith J. Roberts

**Affiliations:** ^1^Department of Trauma and Orthopaedics, University College Hospital, London, NW1 2BU, UK; ^2^The Liver Unit, University Hospitals Birmingham, Birmingham B15 2WB, UK

## Abstract

Assessing a patient's competence to give informed consent in pre-hospital care is difficult. In the presented case an elderly patient attempted suicide by division of a chronically infected and externalised prosthetic arterial graft. He was able to comprehend his situation and understand the consequences of declining treatment. Without prior knowledge of his medical care and psychological state, however, we did not believe we could fully assess the patient's ability to act in his own best interest. After sedation and resuscitation he was transferred to hospital. This case report discusses a unique method of suicide and the challenge of obtaining valid consent in prehospital care.

## 1. Introduction

We report a unique method of suicide by incision of an exposed arterial graft in a housebound octogenarian and the difficult process of dealing with a patient refusing life-saving treatment in the prehospital environment. 

## 2. Case Report

**Figure 1:**
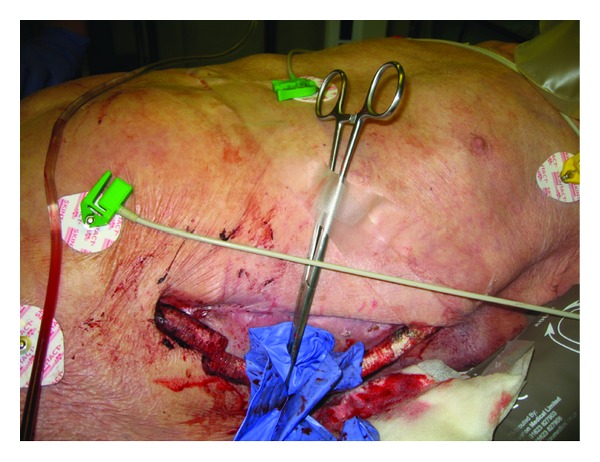
Partially exposed left axillofemoral graft partially divided (arrow) and proximal clamp.

A doctor-led air ambulance team attended an 86-year-old Caucasian male following attempted suicide by an abdominal stab wound. On assessment he was fully conscious, lying in a pool of blood, with a respiratory rate of 35, heart rate of 100, and systolic blood pressure of 60 mmHg. A chronically infected exposed vascular graft had been almost completely divided (left axillofemoral prosthetic graft, Figure 1). His legs were cold, pale, and mottled. Blood clot had formed at the site of injury, but following fluid resuscitation, the increase in blood pressure resulted in further bleeding controlled by placement of a proximal Spencer Wells clamp. Being a case of controlled rather than uncontrolled haemorrhage, hypotensive resuscitation was not practiced (for review see [1]).

History revealed that the patient was housebound due to severe lower limb claudication. He refused hospitalisation as he believed medical intervention would require limb amputation.

A decision to transfer the patient to hospital was made on the basis of his immediately life-threatening condition in the setting of a lack of capacity to consent to or refuse treatment. This decision was made as although the patient clearly was able to communicate refusal of transfer, this seemed to be based on an assumption that consenting to hospitalisation would inevitably lead to further operation including limb amputation. This was despite repeated attempts by ambulance crew members to reassure him that transfer would be provided to allow for better assessment of his condition, adequate resuscitation, and analgesia and only then would further management be considered and discussed with him. He had no next of kin to discuss this with, and his general practitioner was unknown to us. 1 mg boluses of midazolam were used to sedate and alleviate anxiety during transfer and fluid resuscitation continued into hospital. Assessment by vascular, anaesthetic, and intensive care consultants concluded that the patient was very unlikely to survive surgery. This, along with the patient's prior expressed wishes for no treatment, guided the decision to make no further attempts at resuscitation but rather to maintain his comfort. He passed away within three hours.

We subsequently discovered that the left axillofemoral graft replaced an infected right axillofemoral graft which itself replaced an infected abdominal aortic aneurysm repair, complicated by aortoduodenal fistulae. Due to advancing age and frailty, the infected left axillofemoral graft was treated conservatively for 8 years. He had been independent until shortly before his attempt at suicide when he had become housebound.

## 3. Discussion

72% of suicides amongst the elderly are committed by patients with chronic disease [2]. Other risk factors include social isolation and being a white male [2].

Presumably increasing immobility and loss of independence also impacted on this man's decision to attempt suicide. While the most common method of suicide in this age group is by overdose, incision of normal vasculature is relatively rare, comprising only 2–15% of cases [3].

Vascular interventions provide a unique method of suicide with only a small number of cases reported. These include successful suicides by incision of an arteriovenous fistula and a second case involving division of a subclavian venous dialysis catheter [4, 5], both in patients undergoing haemodialysis. This is the first reported case of suicide by incision of a prosthetic vascular graft. Vascular grafts are not uncommon and infection, a recognised complication, is usually treated by antibiotics and either graft replacement, or when exposed, attempts at tissue coverage. This is an extreme case of graft exposure, and it is likely that the externalisation of the graft provided a painless and easy method of suicide.

Refusing to go to hospital because “he did not want to lose his legs” indicates that he was aware that surgery to correct his ischaemic limbs would not be possible as his vascular history and comorbidities clearly precluded attempts to restore low limb perfusion leaving amputation as the only realistic option. We were not aware of his complicated vascular history, but it is now clear that any attempt to restore inflow to his lower limbs would have required complex surgery which he would not have survived. He correctly assumed that division of his graft would lead to inoperable loss of limb perfusion with subsequent death, and this demonstrates insight and a fierce determination not to lose independence.

The decision to take this patient to hospital against his wishes highlights one of the difficulties of working in the prehospital environment. In this case the time-critical nature of the injury and lack of information from secondary parties made assessment of his capacity to make an informed refusal of treatment difficult although it was clear that he was unlikely to survive this episode. Under the Mental Capacity Act 2005, patients should be assumed to have capacity if assessment is not possible. Assessment should establish whether patients can understand, retain, and use information to make a decision regarding treatment and are then able to communicate this [6]. In this case it became clear that the patient did not understand that consent to hospitalisation and consent to operation were separate, and therefore his capacity to refuse consent to hospitalisation alone was judged to be lacking, and in his best interests, sedation and immediate transfer to hospital were undertaken. This allowed expert appreciation of his injuries and assessment of his capacity to consent to or refuse treatment.
